# Genome analysis of *Pseudomonas* sp. OF001 and *Rubrivivax* sp. A210 suggests multicopper oxidases catalyze manganese oxidation required for cylindrospermopsin transformation

**DOI:** 10.1186/s12864-021-07766-0

**Published:** 2021-06-22

**Authors:** Erika Berenice Martínez-Ruiz, Myriel Cooper, Jimena Barrero-Canosa, Mindia A. S. Haryono, Irina Bessarab, Rohan B. H. Williams, Ulrich Szewzyk

**Affiliations:** 1 Chair of Environmental Microbiology, Technische Universität Berlin, Institute of Environmental Technology, Straße des 17. Juni 135, 10623 Berlin, Germany; 2 Singapore Centre for Environmental Life Sciences Engineering, National University of Singapore, Singapore, 119077 Singapore

**Keywords:** Metabolic potential, Manganese-oxidizing bacteria, Biotransformation, Cyanotoxins

## Abstract

**Background:**

Cylindrospermopsin is a highly persistent cyanobacterial secondary metabolite toxic to humans and other living organisms. Strain OF001 and A210 are manganese-oxidizing bacteria (MOB) able to transform cylindrospermopsin during the oxidation of Mn^2+^. So far, the enzymes involved in manganese oxidation in strain OF001 and A210 are unknown. Therefore, we analyze the genomes of two cylindrospermopsin-transforming MOB, *Pseudomonas* sp. OF001 and *Rubrivivax* sp. A210, to identify enzymes that could catalyze the oxidation of Mn^2+^. We also investigated specific metabolic features related to pollutant degradation and explored the metabolic potential of these two MOB with respect to the role they may play in biotechnological applications and/or in the environment.

**Results:**

Strain OF001 encodes two multicopper oxidases and one haem peroxidase potentially involved in Mn^2+^ oxidation, with a high similarity to manganese-oxidizing enzymes described for *Pseudomonas putida* GB-1 (80, 83 and 42% respectively). Strain A210 encodes one multicopper oxidase potentially involved in Mn^2+^ oxidation, with a high similarity (59%) to the manganese-oxidizing multicopper oxidase in *Leptothrix discophora* SS-1. Strain OF001 and A210 have genes that might confer them the ability to remove aromatic compounds via the catechol meta- and ortho-cleavage pathway, respectively. Based on the genomic content, both strains may grow over a wide range of O_2_ concentrations, including microaerophilic conditions, fix nitrogen, and reduce nitrate and sulfate in an assimilatory fashion. Moreover, the strain A210 encodes genes which may convey the ability to reduce nitrate in a dissimilatory manner, and fix carbon via the Calvin cycle. Both MOB encode CRISPR-Cas systems, several predicted genomic islands, and phage proteins, which likely contribute to their genome plasticity.

**Conclusions:**

The genomes of *Pseudomonas* sp. OF001 and *Rubrivivax* sp. A210 encode sequences with high similarity to already described MCOs which may catalyze manganese oxidation required for cylindrospermopsin transformation. Furthermore, the analysis of the general metabolism of two MOB strains may contribute to a better understanding of the niches of cylindrospermopsin-removing MOB in natural habitats and their implementation in biotechnological applications to treat water.

**Supplementary Information:**

The online version contains supplementary material available at 10.1186/s12864-021-07766-0.

## Background

Cylindrospermopsin (CYN) is a secondary metabolite produced by several cyanobacteria, toxic for humans and other living organisms [1]. The two bacterial strains OF001 and A210 transform the cyanotoxin CYN during the oxidation of Mn^2+^ [2, 3]. Strain OF001 belongs to the gammaproteobacteria and was isolated from the effluent of an experimental fixed-bed biofilm reactor established for the removal of recalcitrant substances from wastewater. Strain A210 belongs to the betaproteobacteria and was isolated from an iron manganese-depositing biofilm in a freshwater pond in the Lower Oder Valley National Park, Germany.

The removal of CYN by both strains required the active oxidation of MnCO_3_ whereas no or low CYN removal was observed with MnSO_4_ or in setups without manganese. Sterile biogenic oxides formed by the strains did not show any influence on CYN removal, highlighting the importance of the active manganese oxidation. Both strains are able to remove 100% of CYN at the highest rates reported for biological CYN removal so far [2, 3]. Furthermore, analysis of CYN transformation products revealed that the same seven transformation products were formed by both strains corroborating the important role of manganese oxidation. However, strain OF001 and A210 showed important differences. *Pseudomonas* sp. strain OF001 degraded CYN within 3 days. Whereas strain A210 degraded CYN within 14 to 28 days when cultivated under the same conditions. Moreover, strain OF001 required yeast extract as additional carbon source for the removal of CYN. In contrast, strain A210 was able to transform CYN in mineral media [2].

So far, little is known about biological CYN removal [2–6]. Even though several bacterial strains have been reported to remove CYN, to date, no enzymes or defined metabolic pathway for the transformation of CYN have been identified [7, 8]. Moreover, for biological CYN removal, no transformation products have been identified except for CYN transformed by MOB [3].

MOB are present in terrestrial [9], marine and freshwater environments [10–13], but they also occur in drinking water systems and reactors aiming at the removal of manganese and other pollutants [13–16]. MOB belong to diverse phylogenetic lineages with a broad physiological diversity (e.g. autotrophs and mixotrophs) [10, 17, 18]. Through the oxidation of Mn^2+^, MOB form water-insoluble biogenic manganese oxides, which are one of the strongest natural oxidants [17, 19]. Biogenic manganese oxides often interact with other compounds and thus play an important role in the biogeochemical cycle of manganese and other elements [17, 18, 20, 21].

The physiological role of manganese oxidation is not fully understood. Manganese oxidation was proposed to provide energy to support the growth of bacteria. However, no conclusive results were shown [22]. Other proposed functions are the protection against the toxicity of organic compounds, and reactive oxygen species [23, 24], the breakdown of organic matter into utilizable substrates [25, 26], and the use as a carbon reservoir [27]. Nevertheless, the precise physiological role of manganese oxidation remains unknown [18]. Different manganese oxidation mechanisms have been described including non-enzymatic pathways based on a pH increase, the oxidation through superoxide production, or an anaerobically photo-driven reaction; and enzymatic reactions generally associated to the activity of multicopper oxidases (MCO) and haem peroxidases [11, 18, 28].

Besides CYN, MOB transform different organic and inorganic pollutants, including diclofenac, benzotriazole, 17 α-ethinylestradiol, bisphenol A, As(III), and Sb(III) [2, 3, 29–34]. The mechanism of pollutant transformation was proposed to be based on unspecific oxidation by reactive manganese Mn^3+^/Mn^4+^ species that are formed through the oxidation of Mn^2+^ [29]. For CYN transformation, a similar mechanism was assumed based on the requirement of active Mn^2+^ oxidation for efficient CYN removal and the formation of the same transformation products among all tested MOB, including strain OF001 and A210 [3]. Thus, it is suggested that MOB act as suppliers of biogenic oxides that indirect oxidize the pollutants. However, the intrinsic capacity of MOB to remove organic compounds has not been deeply investigated [18].

The whole genome sequences of some MOB have been analysed previously to gain a better insight into the mechanism of manganese oxidation [35, 36]. However, so far, no reported pollutant-removing MOB strains were analyzed on a genomic level. Besides, for strain OF001 and A210, information about the metabolic potential, including also about genes potentially involved in manganese oxidation, was missing. The genomic analysis of strain OF001 and strain A210 might allow to identify enzymes potentially involved in the oxidation of manganese based on the comparison with manganese oxidizing enzymes reported to date. Furthermore, metabolic differences between the two MOB strains became evident during cultivation experiments in presence of CYN [2], and further dissimilarities could be assumed. Such metabolic differences could be relevant for the application of the strains for the removal of pollutants from water in technical systems including but not limited to wastewater or drinking water treatment plants, and for the understanding of the niche they may occupy in natural environments.

Therefore, in this study, we analysed the draft genomes of the MOB strains OF001 and A210, both of which are able to transform CYN during oxidation of MnCO_3_. We aim to provide further insight into i) manganese oxidation mechanism, ii) other metabolic pathways relevant for pollutant removal, iii) energy harvesting processes such as respiration, iv) their metabolic potential in comparison with their closest described phylogenetic relatives, and v) genome plasticity related to horizontal gene transfer mechanisms.

## Results and discussion

### General genome features

Genome quality estimation determined with CheckM showed that both genomes are of high quality (> 90% completeness and < 5% contamination). Genomes of strains OF001 and A210 have a completeness of 99.59 and 99.38%, respectively, with a contamination level of 2.12 and 0.35%.

The genome sequence of strain OF001 contains 4,476,686 bp in 65 contigs with a N50 contig length of 147,742 bp, and a GC content of 68.01%. The genome of strain OF001 encodes 4845 genes of which 4720 are protein coding sequences (CDS). Furthermore, one 16S, one 23S, and six 5S rRNA genes were identified in the genome of OF001, as well as, sixty-seven tRNA genes that enable recognition of codons for all 20 amino acids.

The genome sequence of strain A210 contains 5,371,534 bp in 72 contigs with a N50 contig length of 327,374 bp, and a GC content of 69.54%. The genome of strain A210 encodes 5184 genes from which 5112 are CDS. In addition, one 5S–23S-16S rRNA operon, and 52 tRNA genes were identified in the genome of strain A210. Genome quality estimation and general genomic features are summarized in Table 1.

**Table 1:** Genomic features of strains OF001 and A210

	OF001	A210
N50	147,742	327,374
Number of contigs	65	72
CheckM completeness	99.59%	99.38%
CheckM contamination	2.12%	0.35%
Complete genome size (bp)	4,476,686	5,371,534
Undetermined bases	6400	7100
% GC	68.01	69.54
% Protein coding density	90.64	93.09
Pseudogene	6	2
CDS	4720	5112
Genes assigned to:		
COG	3436 (72.70%)	3798 (74.27%)
KEGG	2490 (52.7%)	2632 (51.5%)
Hypothetical proteins / unknown function	1327 (28.08%)	1492 (29.17%)
Fragment CDS	6	2
tRNA	67	52
rRNA	8	3
misc_RNA	43	14
tmRNA	1	1

*CDS* Coding sequences, *COG* Cluster of Orthologous Groups, *KEGG* Kyoto Encyclopedia of Genes and Genomes, *tRNA* transfer RNA, *rRNA* Ribosomal RNA, *misc_RNA* Miscellaneous RNA, *tmRNA* Transfer-messenger RNA

Genome Taxonomy Database tool kit (GTDB-tk) was used to classify the bacterial genomes. GTDB-tk analysis classified strain OF001 as a member of the *Pseudomonas*_K group. According to the GTDB (May, 2020) *P. oryzae*, *P. sagittaria*, *P. linyingensis*, and *P. guangdongensis* belong to the *Pseudomonas*_K group. The genus status of the strain OF001 in the *Pseudomonas*_ K group was supported by the genetic relatedness determined by whole-genome analysis and 16S rRNA phylogeny (Additional file 1: Fig. S1).

**Fig. 1:**
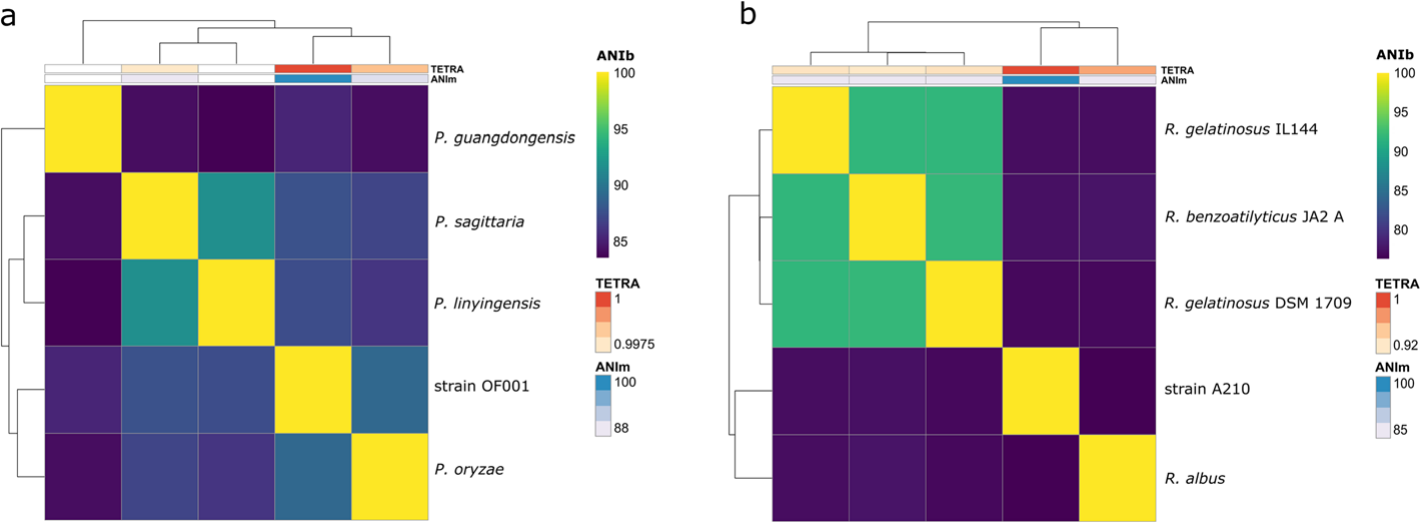
Heatmap representing the degree of similarity of the MOB genomes. **a** *Pseudomona*s sp. OF001, and **b** *Rubrivivax* sp. A210. Heatmaps were derived from the average nucleotide identity (ANI) matrix based on BLAST (ANIb). Dendrogram directly reflects the degree of identity between genomes. ANIm: ANI based on MUMmer; TETRA: correlation indexes of the tetra-nucleotide frequencies; DDH *d*_4_: DDH calculated with the formula *d*_4_, which is the non-logarithmic version of formula *d*_5_ (used for the Fig. S1 and S2). Formula *d*_4_ is highly recommended when using draft genomes to assure confident results [37]

To determine the species affiliation of *Pseudomonas* sp. OF001 average nucleotide identity (ANI), and tetra-nucleotide frequencies (TETRA) analysis were done. The analysis revealed highest similarity between strain OF001 and *P. oryzae* KCTC 32247 with an ANI based on BLAST (ANIb) value of 89.06%, an ANI based on MUMmer (ANIm) value of 90.98%, and a TETRA value of 0.998 (Fig. 1a). Organisms with an ANI value above 95%, and a TETRA value above 0.99 are suggested to delineate the same species level [38–40]. TETRA values should be in agreement with ANI values to support the species assignation [39]. TETRA values of strain OF001 and the organisms of the *Pseudomonas*_K group were higher than 0.99, but ANI values were below the species limit. Together, the data suggest strain OF001 is a potential new species of the *Pseudomonas_*K group.

GTDB-tk analysis classified strain A210 as a member of the *Rubrivivax* genus. According to the GTDB database this genus belongs to the order Burkholderiales and has so far only three described species: *R. benzoatilyticus*, *R. gelatinosus, and R. albus* [41–43].

Based on the phylogenetic analysis using the whole 16S rRNA gene sequence (Additional file 1: Fig. S2a), strain A210 could not be classified at genus level. Organisms with high similarity to the 16S rRNA gene sequence of strain A210 were mainly bacteria of the *genera incertae sedis* from the *Comamonadaceae* family (*Aquabacterium, Ideonella, Leptothrix, Roseateles, Rubrivivax, Sphaerotilus*). However, phylogenomic analysis of strain A210 done with TYGS platform affiliated A210 with organisms of the genus *Rubrivivax* (Additional file 1: Fig. S2b), supporting the results obtained with the GTDB-tk.

ANI, and TETRA analysis were done with the genome of A210 to analyze species affiliation. The analysis showed the highest similarity of *Rubrivivax* sp. A210 with *R. benzoatilyticus* JA2 with an ANIb value of 76.69%, an ANIm value of 84.45%, and a TETRA value of 0.913 (Fig. 1b). Thus suggesting, strain A210 is a potential new species of the genus *Rubrivivax*.

### Pan and core genome

The pan-genome of the *Pseudomonas_*K group genomes comprised 20,296 genes belonging to 6805 Microscope gene Families (MICFAM) [44, 45]. The core-genome comprised 11,985 genes that correspond to 1957 MICFAM, and the variable-genome contained 8311 corresponding to 4848 MICFAM. The *Pseudomonas* sp. OF001 genome contains 1091 strain-specific genes from 1052 MICFAM that correspond to 24.08% strain-specific coding sequences. With this, strain OF001 contains the highest number of CDS from the *Pseudomonas_*K group genomes analyzed (Additional file 1: Fig. S3a). Among the strain-specific genes in OF001, we found genes related to mercury resistance, transport, and foreign DNA (see also section *2.5 Elements potentially acquired by horizontal gene transfer*).

Pan- and core-genome size evolutions were estimated with the four available genomes of the *Pseudomonas_*K group and the genome of strain OF001. The curve of the pan-genome of strain OF001 and *Pseudomonas_*K group did not reach the plateau, suggesting that the pan-genome of *Pseudomonas_*K group is open and the sequences of other genomes from this group might increase the gene pool of novel genes (Additional file 1: Fig. S4a). The plateau of the core-genome is reached with the five genomes selected and is composed of approximately 2000 MICFAM (Additional file 1: Fig. S4b).

The pan-genome of *Rubrivivax* genomes comprised 23,140 genes belonging to 9974 MICFAM. The core-genome comprises 10,154 genes that correspond to 1629 MICFAM, and the variable-genome contains 12,986 genes corresponding to 8345 MICFAM. The *Rubrivivax* sp. A210 genome contains 2123 strain-specific genes from 2035 MICFAM that correspond to 42.98% strain-specific coding sequences (Additional file 1: Fig. S3b). Among the strain-specific genes in A210, we found genes related to transport like ABC transporters, and cytochromes (see also section *2.4.3 Aerobic respiration*).

Pan- and core-genome size evolutions were estimated according to the genomes selected for the A210 analysis (Additional file 1: Fig. S4c-d). The core-genome plateau is apparently reached with the analyzed genomes and is composed of approximately 1600 MICFAM.

### Genes potentially involved in manganese oxidation

**Fig. 2:**
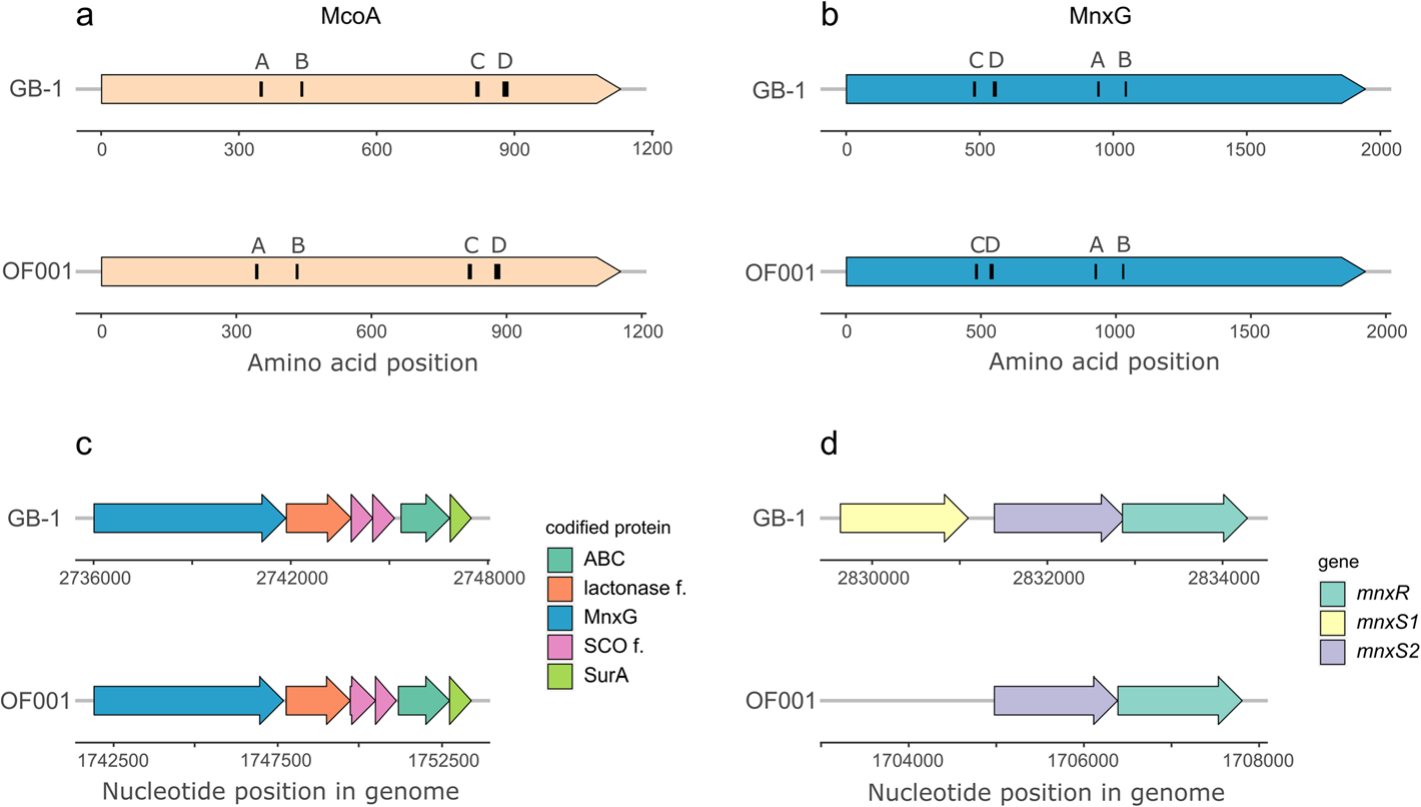
Genetic organization of regulatory system and MO-mco’s in *P. putida* GB-1 and *Pseudomonas* sp. OF001. **a** McoA protein of strain GB-1, and the putative homolog found in strain OF001, **b** MnxG protein of strain GB-1, and the putative homolog found in strain OF001, **c** predicted operon organization in which *mnxG* (MO-mco) is found in strain GB-1, and putative homologues found in a predicted operon in strain OF001, and **d** regulatory system for Mn^2+^ oxidation of strain GB-1, and putative homologues found in strain OF001. Capital letters (A-D) in **a**), and **b**) represent the multicopper oxidase motifs [46]. *mnxR*: response regulator; *mnxS1* and *mnxS2*: sensor histidine kinases; ABC: ABC transporter; lactonase f.: beta-propeller fold lactonase family protein; *mnxG*: MO-mco, SCO f. SCO family protein; SurA: SurA N-terminal domain-containing protein, McoA: MO-mco, MnxG: MO-mco

In *Pseudomonas* sp. OF001, we detected three different homologues of manganese-oxidizing multicopper oxidases (MO-mco’s) (OF001_u20185, OF001_u60094, and OF001_u90046). Gene name, accession number, locus tag in the evaluated genomes, E-value, and percent similarity of amino acid alignments are shown in Additional file 1: Table S1. All three MO-mco’s homologues of strain OF001 belong to the homologous cupredoxin superfamily (IPR008972), according to the InterPro-based analysis. The amino acid sequences encoded by OF001_u20185 and OF001_u60094 exhibit the four characteristic motifs found in multicopper oxidases, in the same order and in a similar position as observed in McoA and MnxG from *P. putida* GB-1 (Fig. 2a, b). In addition, MO-mco’s homologues OF001_u20185 and OF001_u60094, showed the highest similarity to the Mn^2+^ oxidases *mnxG (80%)* and *mcoA (83%)* from *P. putida* GB-1 [47], whereas OF001_u90046 showed the highest similarity to *moxA* (51%) from *Pedomicrobium* sp. ACM 3067 [48]. According to the InterPro-based analysis, all three MO-mco’s contain non-cytoplasmic domain regions of membrane-bound proteins that cover more than 94% of the whole protein sequence. These regions are predicted to be outside the membrane in the extracellular region. Moreover, OF001_u90046 and OF001_u60094 contain transmembrane helixes. The presence of non-cytoplasmic and transmembrane domains suggests that these enzymes are loosely bound to the outer membrane, which is in agreement with the localization of MO-mco’s in other MOB [47, 49, 50]. Functional domains of the proteins and the ontology classification are shown in Additional file 1: Table S2.

OF001_u60094 in *Pseudomonas* sp. OF001 is located in a predicted operon similar to *mnxG* in *P. putida* GB-1 [51]. The operon is composed out of five additional genes with high similarity to those located in the *mnxG* operon of *P. putida* GB-1, (63–76% according to blastp analysis, Fig. 2c). Expression of the MO-mco’s in *P. putida* GB-1 is regulated by a two-component pathway, *mnxS1*/*mnxS2*/*mnxR* [51]. In the genome of strain OF001, we found putative homologues to the *mnxS2* histidine kinase, and to the *mnxR* regulator, arranged in a similar operon structure as in *P. putida* GB-1 (Fig. 2d) [51]. Our results suggest that the regulation of the MO-mco’s of strain OF001 follows a similar regulation to the one observed in *P. putida* GB-1.

Furthermore, two homologues of manganese-oxidizing haem peroxidases (MO-hpox’s) (OF001_u100035, and OF001_u220048) were identified in strain OF001. The putative MO-hpox homologue OF001_u100035 showed the highest similarity with the Mn^2+^ oxidase *mopA* of *P. putida* GB-1 (42%). Together with the MO-hpox of *A. manganoxydans* SI85-9A1, they belong to the haem peroxidase superfamily (IPR010255). The MO-hpox homologue OF001_ u220048, showed highest similarity to *mopA* of *Erythrobacter* sp. SD-21 (38%) and neither of the two belong to the haem peroxidase superfamily (Additional file 1: Table S2). No cytoplasmic or non-cytoplasmic domains could be identified for the putative MO-hpox’s homologues of strain OF001. Therefore, we evaluated the probable subcellular localization with LocTree3 [52]. According to this analysis, both putative MO-hpox’s of OF001 are likely secreted to the media (accuracy percentage 88%), similar as previously described for several MO-hpox’s of other MOB [47–50, 53, 54].

In the genome of *Rubrivivax* sp. A210, five MO-mco’s homologues were identified (RA210_u420004, RA210_u30250, RA210_u110082, RA210_u10102, and RA210_u100111) (Additional file 1: Table S1). Two MO-hpox’s homologues (RA210_u10091, and RA210_u140033) were identified, but were discarded for further analysis due to very low coverage of the query sequences (Additional file 1: Table S1).

**Fig. 3:**
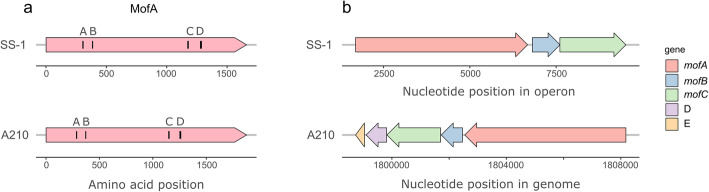
Genetic organization of the MO-mco in *L. discophora* SS-1 and *Rubrivivax* sp. A210. **a** MofA protein of strain SS-1, and the putative homolog found in strain A210, and **b** predicted operon organization in which *mofA* (MO-mco) is found in strain SS-1, and the putative homologues found in a predicted operon in strain A210. Capital letters (A-D) in a) represent the multicopper oxidase motifs [46]. *mofA*: MO-mco; *mofB*: macrophage infectivity potentiator (mip); *mofC*: Cytochrome c domain-containing protein. Note that in **a** the operon in strain SS-1 is represented based on the total length of the operon because the genome has not been sequenced. Capital letters in **b** are the other two proteins predicted within the operon of strain A210, D: copper metallochaperone, and E: protein of unknown function

All MO-mco’s homologues of strain A210 belong to the homologous cupredoxin superfamily (IPR008972), according to the InterPro-based analysis. They contain non-cytoplasmic domains which cover more than 84% of the whole protein sequence, and possess either a transmembrane domain or a transmembrane helix, except for RA210_ u420004. This suggests that these enzymes are loosely bound to the outer membranes, similar as previously reported for other MO-mco’s [47, 49, 50]. RA210_u30250 shows highest similarity (59%) to the *mofA* gene of *L. discophora* SS-1 [49]. In addition, the amino acid sequence of RA210_u30250 encodes the four characteristic motifs found in multicopper oxidases in the same order and in a similar position than those found in MofA from *L. discophora* SS-1 (Fig. 3a). In addition, it is located in a predicted operon similar to *mofA* in *L. discophora* SS-1 (Fig. 3b). The *mof* operon in *L. discophora* SS-1 is composed out of *mofA*, *mofB* and *mofC* [55]. The putative *mof* operon in strain A210 encodes five genes, including the putative *mofA* homologue, and two genes with high similarity to *mofB* (68%) and *mofC* (60%), together with a putative metallochaperon, and an exported protein of unknown function (RA210_u30246 – RA210_u30250).

**Fig. 4:**
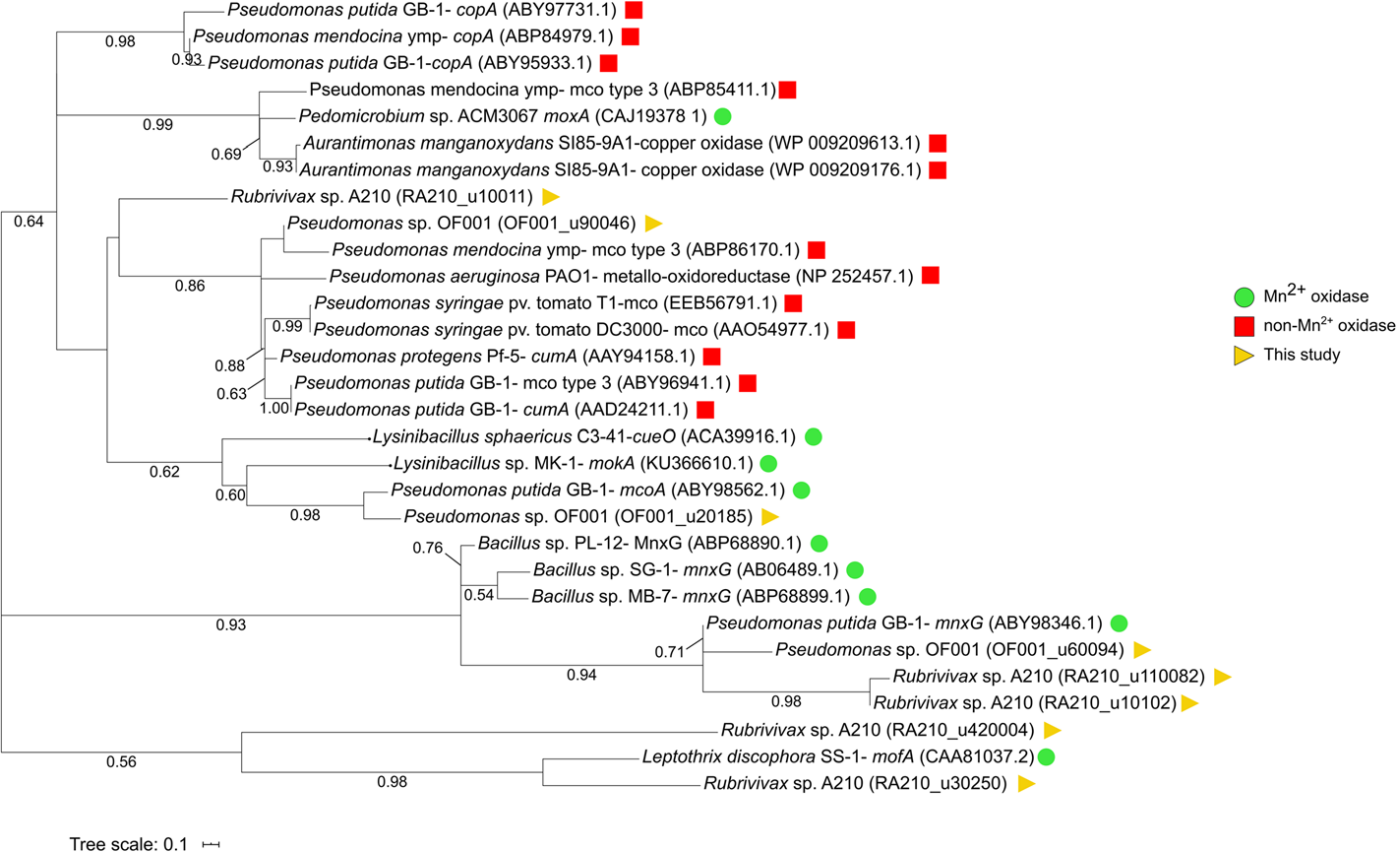
Maximum Likelihood phylogenetic tree based on multicopper oxidase sequences with and without reported Mn^2+^ oxidation activity. Numbers in the branches represent bootstrap values. Scale bar represents sequence divergence

In spite of the low homology between MO-mco’s from different organisms, we attempted to gain further evidence for the Mn^2+^ oxidation activity of the suggested multicopper oxidases by using a phylogenetic approach. For this purpose, a phylogenetic tree was constructed with sequences of MO-mco and non-MO-mco retrieved form the NCBI database excluding the newly identified putative MO-mco homologues (Additional file 1: Table S3), to discard the possibility that the new sequences were the main factor driving the topology of the tree (Additional file 1: Fig. S5). Subsequently, the putative MO-mco homologues of the strains OF001 and A210 were added. Phylogenetic analysis revealed one cluster of all MO-mco sequences and one cluster of non-MO-mco (Fig. 4). The only identified outlier was *moxA* from *Pedomicrobium* sp. ACM 3067, a reported MO-mco, affiliated with the non-MO-mco. Possibly, this is due to an uncertain assignation as suggested previously by Anderson et al. (2009). In contrast to OF001_u20185, OF001_u60094, and RA210_u30250, the proteins encoded by OF001_u90046, and RA210_u100111 did not cluster with the MO-mco (Fig. 4). This result suggests that the two annotated multicopper oxidases OF0011_u90046 and RA210_u100111 in *Pseudomonas* sp. OF001 and *Rubrivivax* sp. A210, respectively, do not possess the Mn^2+^ oxidation activity. Collectively, the data suggest that the best candidates for Mn^2+^ oxidation are MO-mco OF001_u20185, OF001_u60094, and MO-hpox OF001_u100035 in strain OF001 and MO-mco RA210_u30250 in strain A210.

Our results indicate that both MOB strains, OF001 and A210, oxidize manganese through enzyme-mediated mechanisms. In spite of the evidences found based on the genomic analysis, further experiments are required to determine which enzymes are involved in the oxidation of Mn^2+^ in *Pseudomonas* sp. OF001 and *Rubrivivax* sp. A210.

### General metabolism

#### Organic carbon metabolism

*Pseudomonas* sp. OF001 and *Rubrivivax* sp. A210 possess all genes necessary for commonly found central carbohydrate metabolism in aerobic organism including glycolysis (Embden-Meyerhof-Parnas), gluconeogenesis, tricarboxylic acid cycle (Krebs cycle), and the non-oxidative branch of the pentose phosphate pathway, to support basic growth. In both MOB, genes involved in the oxidative branch of the pentose phosphate pathway were incomplete which is in accordance with its absence in many aerobic and thermophilic organisms [56].

#### CO_2_ fixation

*Pseudomonas* sp. OF001 possesses several genes encoding enzymes related to CO_2_ fixation via the Calvin cycle, however the key enzyme D-ribulose-1,5-bisphosphate carboxylase/oxygenase (RuBisCO) is missing (Additional file 1: Table S4). This is in accordance with our previous study which demonstrated the growth of strain OF001 only in presence of an organic carbon source [2].

In contrast, *Rubrivivax* sp. A210 has the complete repertoire of genes required for CO_2_ fixation via the Calvin cycle, including the RuBisCO, which is supported by previous studies of our group showing that A210 was able to grow in mineral media [2]. The *cbb* operon in strain A210 has all genes predicted to be encoded together with the RuBisCO small (*cbxSP*) and large (*cbbL*) subunits (*gpx*, *cbbYP, prkB, fbp, cbxXC*). The presence of genes coding for enzymes of the Calvin cycle in strain A210 is in accordance with their detection in the three described species of the *Rubrivivax* genus *R. albus* [42]*, R. gelatinosus* [43] and *R. benzoatilyticus* [41].

#### Aerobic respiration

All genes for oxidative phosphorylation and aerobic respiration were present in *Pseudomonas* sp. OF001. Among them, twenty genes annotated as cytochromes, including 14 *c*-type, 5 *b*-type, and 1 *d*-type cytochromes were found (Additional file 1: Table S5). Also, several predicted terminal oxidases are present including cytochrome *bd*-type quinol oxidase, cytochrome *c* oxidases, and *cbb*_3_-type cytochrome *c* oxidases.

*cbb*_3_-type cytochrome *c* oxidases in the genome of strain OF001 are predicted to be organized in two operons, one operon containing *cbbP* and *cbbQON* genes, similar as reported for other bacteria [57–60], and one operon containing a copy of *cbbPO* and a gene of unknown function. Next to the *cbbPQON* operon, a predicted operon with three (*ccoSIG*) genes encoding the enzymes responsible of the assembly of *cbb*_3_-type cytochrome *c* oxidases was observed [61]. The *ccoH* assembly factor for the *cbb*_3_-type cytochrome oxidase is missing in strain OF001, suggesting that it follows a *ccoH*-independent assembly mechanism, similar as described for *H. pylori*, and *R. gelatinosus* (Durand et al. 2018).

Likewise, all genes for oxidative phosphorylation and aerobic respiration were found in *Rubrivivax* sp. A210. Thirty-nine genes were annotated as cytochromes, including thirty *c*-type, and nine *b*-type cytochromes. Predicted terminal oxidases are present, including cytochrome *c* oxidases and *cbb*_3_-type cytochrome *c* oxidases (Additional file 1: Table S5). *cbb*3-type cytochrome *c* oxidases and the enzymes responsible of their assembly in the genome of strain A210 are predicted to be organized in a single operon *ccoISNOQPG*. Similar as for OF001, the *ccoH* assembly factor for the *cbb*_3_-type cytochrome oxidase is missing in strain A210, which suggest that it also follows a *ccoH*-independent assembly mechanism likewise to strain OF001.

The presence of diverse cytochrome oxidases, with high O_2_ affinity, rather than only cytochrome *c* oxidases, indicate the potential of strain OF001 and A210 to grow under a wide range of O_2_ concentrations.

#### Nitrogen metabolism

*Pseudomonas* sp. OF001 possesses genes predicted to participate in ammonium uptake, including specific transporters like *amtB* and genes involved in the regulation of the process such as *glnA*, *glnL*, and *glnK* [62–67]. The genes are predicted to be arranged within different operons, with *glnA* as a single regulated gene, located immediately downstream from the operon encoding *glnG* and *glnL* (Additional file 1: Table S4).

In addition, the genome of *Pseudomonas* sp. OF001 encodes the genes *nifDKH*, implicated in nitrogen fixation. Nitrogenase genes in strain OF001 are not predicted to form an operon, but cluster together in the genome. The detection of the *nifDKH* genes is in accordance with their detection in the genomes of the two taxonomically closest organisms to OF001, *P. linyingensis* [68] and *P. sagittaria* [69].

Furthermore, strain OF001 encodes genes related with assimilatory nitrate reduction, including nitrate transport, the ammonium-forming nitrite reductase small subunit *nasD*, and the nitrate reductase *nasA* [70–73]. Strain OF001 also possesses two nitrite reductases, one in a predicted operon together with the nitrate reductase *nasA*, and the other as an independent regulated gene (Additional file 1: Table S4). Genes involved in dissimilatory nitrate reduction were missing. This is in agreement with the absence of genes involved in dissimilatory nitrate reduction in the closest related *Pseudomonas_*K group.

Similar to strain OF001, the genome of *Rubrivivax* sp. A210 encodes genes predicted to participate in ammonium uptake in five predicted operons, including specific transporters like *amtB* and genes involved in the regulation of the process such as *glnA*, *glnL,* and *glnK* [62–67] (Additional file 1: Table S4). However, in contrast to strain OF001, the *glnB* gene is encoded in the genome of strain A210. GlnB is a PII signal transcription protein, homologue to GlnK [74]. Both are key for the metabolic regulation of ammonium uptake. The presence of the *glnK* and *glnB* genes in *Rubrivivax* sp. A210 suggests that ammonium uptake in strain A210 follows a similar regulation as described for *Escherichia coli* [64, 75]. GlnB found in Proteobacteria is commonly associated with glutamine synthetase genes [76], and likewise, the *glnB* gene of strain A210 is located in an operon structure next to the glutamine synthetase *nadE*.

The genes *nifDKH*, implicated in nitrogen fixation, are encoded in the genome of *Rubrivivax* sp. A210 similarly as observed for all three known *Rubrivivax* species [41–43]. The *nifDKH* genes are located in a predicted operon structure together with a putative ferredoxin and a conserved protein of unknown function. Ferredoxin may mediate nitrogenase activity when ammonium is available for uptake [77–80].

Moreover, strain A210 encodes genes related with assimilatory nitrate reduction, including nitrate transport, the ammonium-forming nitrite reductase small subunit *nirB*, and the nitrate reductase *nasA* [70–73].

In contrast to strain OF001, in the genome of strain A210 genes related with dissimilatory nitrate reduction were detected, including nitrate reductase *narGHI*, and nitrite reductase *nirBD.* Noteworthy, in the three described species of the *Rubrivivax* genus [41–43]*,* genes coding for enzymes related to dissimilatory nitrate reduction were not detected.The enzymes related with the dissimilatory and assimilatory nitrate reduction are organized in two predicted operons.

The genomic data indicate that both MOB strains have the ability to assimilate ammonium. In addition, it seems likely that OF001 can use nitrate in an assimilatory but not dissimilatory pathway. In contrary, the genomic data suggest that *Rubrivivax* sp. A210 has not only the genomic potential to assimilate nitrate, but also to perform anaerobic respiration using nitrate as final electron acceptor. This characteristic may confer a higher flexibility to strain A210, compared to OF001, to adapt to changing conditions in technical and natural environments.

#### Sulfur metabolism

*Pseudomonas* sp. OF001 harbours all genes required for assimilatory sulfate reduction, which are organised in several predicted operons, and some as single regulated genes (Additional file 1: Table S4). Strain OF001 also possesses different sulfate transporters including ABC type UWA [81], the proton: sulfate symporter or putative sulfate: bicarbonate antiporter SulP [82, 83], and the high affinity sulfate transporter CysZ, essential for sulfate uptake at low concentrations [84, 85]. No genes required for dissimilatory sulfate reduction were detected in the genome of strain OF001.

*Rubrivivax* sp. A210 harbours several genes required for assimilatory sulfate reduction, organised in three predicted operons (Additional file 1: Table S4). However, A210 lacks the adenylyl sulfate kinase *cysC*, responsible of the transformation of adenosine 5′-phosphosulfate (APS) to 3′-phosphoadenosine-5′-phosphosulfate (PAPS), which is an essential step in the assimilatory sulfate reduction [86]. Nevertheless, other organisms like *P. aeruginosa*, *Sinorhizobium meliloti*, and *Burkholderia cenocepacia* lacking *cysC*, reduce APS via the phosphoadenosine phosphosulfate reductase *cysH* to sulphite [87–89]. Strain A210 also possesses different sulfate transporters like ABC type UWA [81]. Similar to strain OF001 and other *Rubrivivax* species, not all genes involved in dissimilatory sulfate reduction were identified in strain A210.

#### Iron metabolism

*Pseudomonas* sp. OF001 possess genes predicted to participate in the transport and storage of iron, including ferrous and ferric iron transporters and bacterioferritin [90] (Additional file 1: Table S4). Strain OF001 also possesses different genes related to heme uptake, such as heme-binding protein and periplasmic heme chaperone [91]. AntiSmash analysis could not detect gene clusters related to siderophores synthesis in strain OF001 genome. Nonetheless, the search with blastp revealed the presence of thirty-one genes related to the synthesis and transport of siderophores. Twenty-one out of the thirty-one genes detected in the genome of strain OF001, correspond to siderophores transport (Additional file 1: Table S4).

Similar to strain OF001, the genome of *Rubrivivax* sp. A210 encodes genes predicted to participate in the transport and storage of iron, such as ferric iron transporters and bacterioferritin [90] (Additional file 1: Table S4). Strain A210 also possesses different genes related to heme uptake, such as heme-binding protein and periplasmic heme chaperone [91]. AntiSmash could not detect gene clusters related to siderophores synthesis in strain A210 genome. However, the search with blastp revealed the presence of thirty-six genes related to the synthesis and transport of siderophores. Twenty-two out of the thirty-six genes detected in A210 genome, correspond to siderophores transport (Additional file 1: Table S4).

#### Cell motility and biofilm formation

Proteins for motility, including genes related to chemotaxis, and flagellar proteins were present in the genome of *Pseudomonas* sp. OF001 (Additional file 1: Table S6). Genes encoding flagellar proteins in strain OF001 belong to the *flg*, and *fli* family, which are part of the core set of flagellar genes [92]. Among the genes related to the central signal transduction pathway for chemotaxis, we found *cheAWYBR* genes, and the transmembrane chemoreceptors, methyl-accepting chemotaxis proteins (MCPs) in the genome of strain OF001. This is in agreement with the description of the closest relatives of strain OF001, *P. oryzae*, *P. sagittaria*, *P. guangdongensis*, and *P. linyingensis*, as motile bacteria [68, 69, 93, 94]. Sixty-four genes associated with biofilm formation were found in the genome of *Pseudomonas* sp. OF001, including *siaD*, *bifA*, and *fleQ* (Additional file 1: Table S6).

In the genome of *Rubrivivax* sp. A210 genes required for motility were present, including genes related to chemotaxis, and genes encoding flagellar proteins (Additional file 1: Table S6). Similar as in strain OF001, in strain A210 found genes encoding flagellar proteins belong to the *flg*, and *fli* family. Strain A210 possesses *cheAWYBR* genes, and the transmembrane chemoreceptors MCPs, which are related to the central signal transduction pathway for chemotaxis. The taxonomically closest bacteria to strain A210, *R. gelatinosus,* and *R. benzoatilyticus*, are also motile bacteria [41, 43]. However, despite the presence of motility-related genes in *R. albus*, the absence of motility was experimentally evidenced [42]. Therefore, further experiments are required to verify motility of strain A210. One hundred twenty one genes associated with biofilm formation were found in the genome of *Rubrivivax* sp. A210, including *sadC*, *pilI*, and *pslH*. (Additional file 1: Table S6).

As discussed above, both strains contain genes that encode proteins involve in biofilm formation, which is in agreement with the isolation of both MOB from biofilms. Moreover, both strains form biofilm in pure culture.

#### Organic compound degradation

Genes for the aerobic degradation of aromatic compounds via the catechol meta-cleavage pathway, and for the specific degradation of benzoate, phenol, and benzene, including phenol/toluene 2-monooxygenases (NADH), benzoate/toluate 1,2-dioxygenases, and catechol 2,3-dioxygenases, were detected in *Pseudomonas* sp. OF001 (Additional file 1: Table S7). In addition, strain OF001 may have the potential to transform other compounds like 2-, 3- and 4-fluorobenzoate, toluene, steroids, citalopram, trinitrotoluene, p-methylbenzoate, trans-cinnamate, phenylpropanoate, and 4-hydroxyphenylacetate. This is in accordance with the ability of several *Pseudomonas* spp. to transform diverse organic pollutants such as benzoate, toluene, phenol, and poly- chlorobiphenyls (PCBs) [95].

Because strain OF001 is able to degrade the cyanotoxin CYN we were also interested in the potential of the strains to degrade other cyanobacterial toxins. However, specific enzymes for the degradation of cyanotoxins are described only for microcystin, the most studied cyanotoxin [7, 8]. No genes involved in microcystin degradation were found in the genome of OF001. Although biodegradation of CYN is considered one of the main natural attenuation processes [96], no specific genes involved in their transformation are known yet [3].

*Rubrivivax* sp. A210 harbors genes involved in the aerobic degradation of aromatic compounds via the catechol ortho-cleavage pathway, and for the specific degradation of benzoate, and 3- and 4-fluorobenzoate, including benzoate/toluate 1,2-dioxygenases, muconate cycloisomerase, and catechol 1,2-dioxygenase (Additional file 1: Table S7). Similarly, the closely-related strain *R. bezoatilyticus* JA2 catabolizes different aromatic compounds including benzoate [41]. Strain A210 also has the potential to transform other compounds such as 4-methylcatechol, acrylonitrile, 2-fluorobenzoate, and trinitrotoluene.

Because strain A210 is able to degrade CYN similarly as strain OF001, we searched the genome for genes related to cyanotoxin transformation. We could not find genes associated with the transformation of microcystin.

### Elements potentially acquired by horizontal gene transfer

#### Genomic islands

Genomic islands are genomic regions potentially obtained by horizontal gene transfer that can drive strain differentiation and support adaptation. Analysis of *Pseudomonas* sp. OF001 genome with IslandViewer 4 led to the identification of at least 12 genomic islands with size ranges from 4.2 to 70.5 Kb (Additional file 1: Table S8 and Fig. S6a). Genomic islands in strain OF001 include genes associated with transposases, phage proteins, CRISPR systems, 147 proteins of unknown function, toxin-antitoxin systems, metal-related proteins, and mercury resistance. Metal resistance genes are related with environmental pollution, and specifically, mercury resistance genes are the genes most frequently associated with genomic islands [97].

Analysis of *Rubrivivax* sp. A210 genome with IslandViewer 4 led to the identification of at least 8 genomic islands with size ranges from 3.8 to 99.5 Kb (Additional file 1: Table S8 and Fig. S6b). Genomic islands in strain A210 included genes associated with transposases, phage proteins, 136 proteins of unknown function, toxin-antitoxin systems, transporters, and nitrate reduction. Genes related to nitrogen metabolism associated to genomic island have been previously reported [98, 99].

#### Prophages

Using PHASTER (PHAge Search Tool Enhanced Release), we detected four incomplete and three intact prophage regions (Score ≥ 100) in *Pseudomonas* sp. OF001 genome (Additional file 1: Table S9 and Fig. S7a). The three intact prophages were named OF001 region 2, OF001 region 5, and OF001 region 7 based on the genome location retrieved by PHASTER. A summary of the distribution and genetic features of these prophages is shown in Additional file 1: Fig. S7b. All prophages in OF001 exhibited structural proteins, including major capsid, fiber, and tail proteins.

Based on the proteomic tree generated with the VIPTree server, all complete prophages in OF001 belong to the order Caudovirales (Additional file 1: Fig. S8). OF001 region 2 and OF001 region 5 were classified in the family Siphoviridae, while OF001 region 7 was classified in the family Myoviridae. Interestingly, OF001 region 2 and OF001 region 7 display putative site-specific integrases and excisionases, indicating site-specific recombination [100]. Multiple prophages have already been observed in other members of the genus *Pseudomonas* [101–104].

Within A210 genome, two incomplete prophages of 28.8 and 9.9 kb were detected (Additional file 1: Table S9 and Fig. S7c). No complete prophage was identified.

### CRISPR-Cas systems

Using the CRISPRCas finder tool, we identified one complete class 1 CRISPR-Cas system, with a level of confidence of 4 (levels from 1 to 4, representing level 4 the most confident identification [105]) in the genome of OF001 (Additional file 1: Table S10 and S11). The seven *cas* genes are downstream of the repeat/spacer region. The repeats and spacers were compared with the CRISPRCas database [106] and were highly similar to sequences found in other bacteria including some Pseudomonads like *P. stutzeri*, *P. aeruginosa*, and *Pseudomonas* sp. phDV1 (Additional file 1: Table S12 and S13). From the taxonomically closest organisms to strain OF001, only *P. guandongensis* has one confirmed class 1 CRISPR/Cas system.

In the genome of A210, one complete class 1 CRISPR-Cas system, with a level of confidence of 4, and one CRISPR region without *cas* genes associated, with a level of confidence of 2 were identified (Additional file 1: Table S10 and S11). The three *cas* genes associated to the complete CRISPR-Cas loci are downstream of the repeat/spacer region. The repeats and spacers of the CRISPR region with a level of confidence 4 were compared with the CRISPRCas database [106] and, for the majority of them, no matching were found (Additional file 1: Table S14 and S15). For the three spacers and two repeats, only results with a similarity around 50% were found. The organisms were these repeats and spacers were found are *Verrucomicrobium spinosum, Raphidiopsis curvata, Pectobacterium carotovorum* and *Opitutaceae bacterium*. The taxonomically closest organisms to strain A210, *R. gelatinosus* and *R. benzoatilyticus*, have two CRISPRs without associated *cas* genes, and 4 incomplete and 2 complete CRISPR-Cas systems, respectively.

Class 1 CRISPR-Cas systems, as the one found in both MOB, are the most abundant class in Beta and Gammaproteobacteria, and in general in archaea and bacteria [107].

The presence of CRISPR-Cas systems in strain OF001 and A210 might represent protection from phage infections, but could represent a disadvantage if useful genes for competitive adaptation cannot be acquired via external DNA [108].

Together the presence of genomic islands, including phage material and CRISPR-Cas systems in *Pseudomonas* sp. OF001 and *Rubrivivax* sp. A210 suggest that both MOB have undergone diverse genetic changes related to different horizontal gene transfer mechanisms which likely contribute to their genome plasticity.

### Implications of the metabolic potential of strains OF001 and A210

In this study, we aimed at a better understanding of the metabolic capacities of the two CYN removing MOB which could potentially contribute to the biotechnological use of MOB for the removal of pollutants from water. In agreement with the genomes of other MOB with so far uncharacterized degradation ability [35, 36], the content of the genomes of strain OF001 and strain A210 suggests a potential metabolic versatility and thus, a broader application potential.

The genomic potential of MOB strains OF001 and A210 for the degradation of different organic compounds via specific enzymatic pathways might complement the unspecific transformation pathways of substances like diclofenac [109] and CYN [2, 3], via manganese oxidation. Our results suggest that strain OF001 and A210 might be able to remove different organic pollutants by a coupled mechanism involving specific enzymatic activity and unspecific oxidation by the reactive manganese species, as has been observed for the removal of phenolic compounds, which are common wastewater pollutants [110]. Moreover, it seems likely that the MOB described in this study transform other organic compounds like carbofuran, ciprofloxacin, and 17α-ethinylestradiol, similar to other MOB [30, 111, 112].

Both analyzed MOB, strain OF001 and A210, transform CYN indirectly through the oxidation of Mn^2+^ [3], and according to the results of this study most likely mediated by the activity of multicopper oxidases and haem peroxidases. The unspecific transformation of CYN by MOB does not require an adaptation phase or a pre-conditioned towards the toxin as it is known for many enzymatically catalyzed processes. Therefore, the use of MOB to remove the only periodically occurring CYN molecule, might represent an advantage in comparison to other biological removal processes that require a pre-conditioning with the toxin to remove it. Moreover, the unspecific oxidation of organic pollutants via reactive manganese species might allow for the removal of other cyanotoxins, however further studies are required.

The different metabolic pathways encoded in the genome of strain OF001 and strain A210 also suggest different fields of application aiming at the removal of pollutants. For instance, the ability of strain A210 to thrive and degrade CYN in the absence of an organic carbon source suggests that it is more suitable for an application in settings, in which readily degradable organic carbon sources are depleted, such as reactors for the removal of pollutants from secondary wastewater. Also, due to the metabolic potential of strain A210, it may adapt within the reactor or the biofilm to varying oxygen concentrations or even the depletion of oxygen by a shift to nitrate respiration [113]. Moreover, both strains are able to form biofilms which may allow them to establish and be retained on fixed bed reactors.

*Pseudomonas* sp. OF001 showed the highest CYN removal efficiency and the fastest growth from all tested MOB [2]. Furthermore, it was isolated from a fixed-bed reactor system, however, the genome of strain OF001 encodes less diverse metabolic pathways to adapt to changing environments. Together, this data suggests that studies investigating degradation potential of MOB should consider the phylogenetic and metabolic diversity of MOB to identify the most suitable organisms that fulfil the requirements of the removal system.

The metabolic diversity of strains OF001 and A210 also suggests an important role of MOB in the removal of CYN in different habitats. For instance, strain A210 was isolated from a freshwater lake in the National Park Lower Oder Valley in Germany. This strain has the metabolic potential to dissimilatory reduced nitrate, which is an important mechanism to control nitrogen loading in aquatic environments [114–116]. Dissimilatory nitrate reduction to ammonium has been related to the promotion of eutrophic conditions in water systems, due to the release of ammonium that could be used preferentially by cyanobacteria, and therefore favouring cyanobacterial blooms [116]. MOB strains with the ability to denitrify and degrade CYN may be therefore tightly interconnected with the production and removal of the cyanotoxin. Furthermore, the metabolic versatility of MOB may allow them to inhabit sediments and water columns. Therefore, MOB might contribute to the removal of CYN produced by benthic organisms in sediments, but also might transform CYN produced by planktonic cyanobacteria in the water column. However, further studies on the occurrence and distribution of MOB in CYN contaminated environments are required.

## Conclusions

In summary, this study provides an insight into the molecular basis of Mn^2+^ oxidation, and into the metabolic potential of two CYN-transforming MOB strains. We identified sequences in *Pseudomonas* sp. OF001 and *Rubrivivax* sp. A210 that show high similarity to already described MCOs which may catalyze manganese oxidation required for CYN transformation. Furthermore, considering the mechanism proposed for the removal of other pollutants by MOB the multicopper oxidases found in both strains and the haem peroxidase identified in strain OF001 might covey the ability to both strains to transform also other pollutants susceptible to reactive Mn species. Both MOB share the potential to grow over a wide range of O_2_ concentrations, to fix nitrogen, and reduce nitrate and sulfate via the assimilatory pathway. Both strains encode pathways that might enable them to remove different aromatic compounds such as benzoate, benzene, and phenol. However, while strain A210 harbors the genomic potential to fix CO_2_ and to reduce nitrate as final respiratory electron acceptor, strain OF001 requires additional organic carbon sources and lacks the ability for dissimilatory nitrate reduction. The analysis of the general metabolism of two MOB strains able to remove organic pollutants such as CYN and DCF might help to implement MOB in biotechnological applications and contributes to a better understanding of the natural niches of CYN-removing MOB in natural habitats.

## Methods

### Strains, culturing conditions and genomic DNA extraction

*Pseudomonas* sp. OF001 and *Rubrivivax* sp. A210 were obtained from the culture collection of the Laboratory of Environmental Microbiology from the TU Berlin, Germany [2]. Bacteria were routinely cultivated in a medium that was originaly developed for *Leptothrix* strains [117], which was modified by our research group and is known as LSM2.

Cells from a pure, fresh 50 mL liquid culture from each strain were harvested by centrifugation at 15,000 x *g* for 3 min and washed three times with sterile Milli Q water under sterile conditions. Total genomic DNA was extracted using the GeneMATRIX Soil DNA Purification Kit (EUR_X_ Gdańsk, Poland) following the manufacturer’s instructions. Quality and quantity of the extracted DNA was determined using QubitTM fluorometric quantitation and NanoDrop 2000 (both Thermo Fisher Scientific, Bremen, Germany).

### Genome sequencing, assembly and annotation

The genome of both MOB strains was sequenced on an Illumina MiSeq platform with a read length of 301 bp (paired end). The genome of each isolate was assembled using SPAdes 3.10.1 and draft genomes obtained using manual binning procedures based on coverage-GC plots performed in R 3.6.1 (Additional file 1: Fig. S9). Genome quality estimation based on completeness and contamination was determined with CheckM [118]. Genome annotation was performed with the interface Magnifying Genomes (MaGE) of the MicroScope web-based service from GenoScope [119]. Protein coding genes were classified based on the annotation into Cluster of Orthologous Groups (COG) functional categories [120] with the automatic classification COG tool at Microscope platform. Function and pathway analysis were performed using BlastKOALA web tool of KEGG (Kyoto Encyclopedia of Genes and Genomes) database according to the KEGG groups of orthologs [121], and using MicroCyc tool of the MicroScope web-based service from GenoScope [119] which is a collection of microbial Pathway/Genome databases (PGDBs). PGDBs within MicroScope are generated by comparing the genome annotations to the metabolic reference database MetaCyc [122]. In the present work, metabolic potential will refer to the possibility of the strains to follow a specific metabolic pathway based only on their genome information, without being so far experimentally corroborated.

The data for this study have been deposited in the European Nucleotide Archive (ENA) at EMBL-EBI under project number PRJEB40009 with accession numbers GCA_904426495 and GCA_904426505 for strain OF001 and A210, respectively (https://www.ebi.ac.uk/ena/browser/view/PRJEB40009).

### Genomes comparison

First classification of the genomes was determined according to the Genome Taxonomy Database (GTDB) using the GTDB-tool kit (GTDB-tk) v.1.1.0 integrated in the MicroScope web-based service [44, 123, 124]. GTDB-tk provides a taxonomic classification of bacterial and archaeal genomes based on the combination of the GTDB reference tree, the relative evolutionary divergence and the ANI value against reference genomes [123]. GTDB proposed a bacterial taxonomy based on the phylogeny inferred from the concatenation of 120 ubiquitous single-copy proteins that normalizes taxonomic ranks by using the relative evolutionary divergence [124]. Therefore, it is considered that the analysis performed by GTDB-tk has an advantage over other phylogenies currently in use [124].

Genomes sequences were uploaded to the Type strain genome server (TYGS), a free bioinformatics platform (https://tygs.dsmz.de) for a whole genome-based taxonomic analysis [125]. TYGS platform runs automatically all the analysis. Briefly, TYGS performed first a determination of closely related type strain genomes, comparing the query genome against all available genomes in the TYGS database with the MASH algorithm [126] and selecting ten type strains. Then, additionally ten close related type strains were determined based on the 16S rRNA sequence extracted from the query genome using RNAmmer [127]. 16S rRNA sequences were compared with BLAST [128] against the TYGS database. The best 50 matching types were used to calculate precise distances using the Genome BLAST distance phylogeny approach (GBDP) [37]. The distances calculated by GBDP were then used to determine the ten closest type strain genomes for each query. Afterwards, GBDP conducted all pairwise comparisons among the set of genomes selected in the previous steps, and inferred accurate intergenomic distances under the algorithm “trimming” and distance formula *d*_*5*_ [37]. One hundred distance replicates were calculated each. In silico DNA-DNA hybridization (DDH) analysis were calculated using the recommended settings of the Genome-to-genome distance calculator (GGDC) 2.1 [37]. The resulting intergenomic distances were used to infer a balanced minimum evolution tree with branch support via FASTME 2.1.4 including subtree pruning and regrafting (SPR) postprocessing [129]. Branch support was inferred from 100 pseudo-bootstrap replicates each.

JSpeciesWS [40] was used to calculate the average nucleotide identity (ANI) values [40] based on BLAST (ANIb) [38, 128] and MUMmer (ANIm) [130], and to calculate the correlation indexes of the tetra-nucleotide frequencies (TETRA) [131].

For the ANI and TETRA analysis, the genome of strain OF001 was compared to the Pseudomonads belonging to the *Pseudomonas_*K group: *P. oryzae* (GCA_900104805.1), *P. sagittaria* (GCA_900109175.1), *P. guangdongensis* (GCA_900105885.1), and *P. liyingensis* (GCA_900115715.1).

For the ANI and TETRA analysis, the genome of strain A210 was compared to the genomes of the three species of the genus *Rubrivivax*: *R. benzoatilyticus* JA2 (GCA_000420125.1), *R. gelatinosus* IL144 (GCA_000284255.1), *R. gelatinosus* DSM 1709 (GCA_00430905.1), and *R. albus* ICH-03 (GCA_004016515.1).

### Core- and pan-genome

Determination of the core- and pan-genome analysis was performed with the Pan/Core-genome tool from the MicroScope web-based service [119]. The analysis is based on the computation of Microscope gene families (MICFAM) using a single linkage clustering algorithm of homologous genes sharing an amino-acid alignment coverage and identity above the defined threshold [45]. This analysis considered i) any MICFAM associated with at least one gene from every genome used for the comparison as a part of the core-genome, ii) any MICFAM associated with at least 2 compared genomes as a part of the variable- genome, and iii) the sum of the core-genome and variable-genome as the pan-genome [44]. Parameter of 50/80 was selected (50% amino-acid identity, 80% amino-acid alignment coverage). All bacterial genomes used for the comparison with the genomes of strain OF001 or strain A210 that were not available in the MicroScope database were also annotated with MaGe from GenoScope [119].

For the pan- and core-genome analysis, the same strains as for the ANI and TETRA analysis, were used.

### Manganese-oxidation genes

We used the blastp function on the Microscope web server [128, 132] to identify potential Mn^2+^ oxidases in *Pseudomonas* sp. OF001 and *Rubrivivax* sp. A210, using experimentally verified Mn^2+^ oxidases of other manganese-oxidizing bacteria. Nine sequences of multicopper oxidases and three sequences of haem peroxidases related with the oxidation of Mn^2+^ in other MOB were used for the search (Table S1). Multicopper oxidases and haem peroxidases with Mn^2+^ oxidation activity will be referred as MO-mco and MO-hpox, respectively. We considered as homologue any protein with an E-value lower than 10^− 10^.

Mn^2+^ oxidases and putative homologues found in OF001 and A210 were functionally analyzed with the InterPro web server [133]. InterPro web server classifies proteins into families, and predicts functional domains and important sites of the proteins, integrating protein signatures from 13 different databases. We predicted the sub-cellular localization with LocTree3 [52] of those putative homologues without a predicted cytoplasmic or non-cytoplasmic domain according to the InterPro analysis.

To determine a possible phylogenetic relationship between manganese-oxidizing multicopper oxidases (MO-mco) and non-manganese oxidizing multicopper oxidases (non-MO-mco), we created a dataset sequences of multicopper oxidases with experimental evidence of Mn^2+^ oxidation [47–49, 53, 134, 135] and multicopper oxidases with experimental evidence of non-Mn^2+^ oxidation activity [47, 54] and included our sequences. They were aligned using MUSCLE [136] in MEGA v7.0.25. A phylogenetic tree was constructed with Maximum Likelihood method in MEGA v7.025. A bootstrap analysis was performed with 1000 replicates for the Maximum Likelihood tree.

### Operon prediction

Operon prediction was done using the FGENESB program [137]. FGENESB gene prediction algorithm is based on Markov chain models of coding regions, start of translation, and termination sites. Predicted genes are then used for the operon models using distances between ORFs frequencies of neighboring genes in known bacterial genomes, and positions of predicted promoters and terminators [137].

### Siderophores

Identification of siderophore biosynthesis gene clusters was performed with AntiSMASH tool [138] from the MicroScope web-based service. In addition, we used the blastp function on the Microscope web server [128, 132] to search for genes previously reported for the biosynthesis of siderophores pyoverdine, enterobactin, yersiniabactin, ornibactin and pyochelin [139–142].

### Elements potentially acquired by horizontal gene transfer

#### Genomic islands

Genomic islands were predicted with the IslandPath-DIMOB [143] and SIGI-HMM [144] method included in the IslandViewer 4 tool using the default settings [145]. Among the prediction methods included in IslandViewer 4 tool, SIGI-HMM has the highest precision and overall accuracy [146].

#### Prophages

Putative phages from *Pseudomonas* sp. OF001 and *Rubrivivax* sp. A210 were predicted with PHASTER (PHAge Search Tool Enhanced Release) web server [147]. PHASTER classifies genome regions with a score below 70 as incomplete, between 70 to 90 as questionable, and greater than 90 as complete prophages [147].

The resulting complete prophage genomes were annotated with multiPhATE v.1.0 (multiple-genome Phage Annotation Toolkit and Evaluator) [148] using Phanotate to predict ORFs [149]. PhAnToMe (Phage Annotation Tools and Methods), pVOGs [150], and SwissProt [151] databases were used for the identification of the homologs of the input genomes and its predicted gene and peptide sequences. Additionally, highly divergent structural proteins were detected with iVireons [152] and confirmed with VIRALPro [153].

To classify the complete prophages, a whole proteomic tree based on genome-wide similarities was computed by tBLASTx, using the VIPTree web server v.1.9 [154].

### CRISPR-Cas systems

The presence of Clustered Regularly Interspaced Short Palindromic Repeats (CRISPR) and their associated genes (*cas*) was evaluated with CRISPRCasFinder [105]. CRISPRCasFinder include a rating system which classifies the detected CRISPRs to differentiate between CRISPR-like elements and true CRISPRs. Evidence levels from 1 to 4 are assigned, with 1 representing the lowest evidence classification and 4 the most confident identification [105].

Spacers and repeat regions detected in both genomes were searched with BLAST (blastn) against the CRISPRCasdb to identify their presence in other organisms. CRISPRCasdb contains CRISPR arrays and *cas* genes from complete genome sequences [106].

### Data graphics

Figures were made with the R packages ggplot2 (Wickham, 2016), gridExtra (Auguie, 2017), pheatmap [155], VennDiagram [156], and gggenes [157], using Viridis [158] and RcolorBrewer [159] packages for colouring in RStudio version 1.0.153 [160, 161]. Genomic maps of prophages were generated using the Snapgene® software (GSL Biotech). Trees generated with the TYGS tool and the multicopper oxidase tree were visualized and annotated with the online server iTOL [162].

## Supplementary Information

**Additional file 1: Fig. S1.** Phylogenetic tree based on 16S rDNA sequences and whole genome sequences including strain OF001 sequence. Tree inferred with FastME 2.1.6.1 [129] from GBDP distances calculated from a) 16S rDNA gene sequences and b) genome sequences. The branch lengths are scaled in terms of GBDP distance formula *d*_5_. The numbers above branches are GBDP pseudo-bootstrap support values > 60% from 100 replications, with an average branch support of a) 68.8% and b) 92.5%. Tree was rooted at the midpoint [163]. Bold text represent the sequences generated in the present work. Scale bar represent sequence divergence. **Fig. S2.** Phylogenetic tree based on 16S rDNA sequences and whole genome sequences including strain A210 sequence. Tree inferred with FastME 2.1.6.1 [129] from GBDP distances calculated from a) 16S rDNA gene sequences and b) genome sequences. The branch lengths are scaled in terms of GBDP distance formula *d*_5_. The numbers above branches are GBDP pseudo-bootstrap support values > 60% from 100 replications, with an average branch support of a) 76.8% and b) 83.4%. Tree was rooted at the midpoint [163]. Bold text represent the sequences generated in the present work. Scale bar represent sequence divergence. **Fig. S3.** Pan- and core genome overview. Venn diagram shows the number of shared and specific Microscope gene families (MICFAM) a) among *Pseudomonas* sp. OF001 and the members of the *Pseudomonas*_K group, and b) among *Rubrivivax* sp. A210 and the members of the *Rubrivivax* genus. MICFAM grouping was based on 50% amino acid identity cut-off and at least 80% amino-acid alignment coverage. **Fig. S4.** Pan- and core- genome sizes estimated evolution. a, c) Number of MICFAM families in the pan-genome size by the number of genomes, and b, d) number of MICFAM families in the core-genome by the number of genomes. a, b) Including *Pseudomonas* sp. OF001, and c, d) including *Rubrivivax* sp. A210. **Fig. S5.** Maximum Likelihood phylogenetic tree based on multicopper oxidases sequences with and without reported Mn^2+^ oxidation activity. Sequences of the studied strains in the present study are not included. Numbers in the branches represent bootstrap value. Scale bar represent sequence divergence. **Fig. S6.** Putative genomic islands harbored by the studied MOB. a) *Pseudomonas* sp. OF001, and b) *Rubrivivax* sp. A210. Outer circle represents the genome size in Mbps. Genomic islands obtained by different prediction methods are highlighted in color. Integrated represent those islands detected by at least one method. **Fig. S7.** Distribution and genetic features of prophages detected in *Pseudomonas* sp. OF001 and *Rubrivivax* sp. A210. a) Circular genome map of strain OF001, b) genetic features of the complete prophages in strain OF001, and b) circular genome map of strain A210. In the genome maps location of prophages are highlighted in colors depending on the completeness of the prophages (Table S9). Number assigned to each prophage region is based on the genome location retrieved by PHASTER [147]. **Fig. S8.** Whole proteomic tree of *Pseudomonas* sp. OF001 prophages based on genome-wide similarities computed by tBLASTx. The tree was constructed using the VIPTree web server v.1.9 [154]. Numbers in brackets in the figure legend represent the number of virus genomes. Red stars represent the three complete prophages of strain OF001. Scale bar represent sequence divergence. **Fig. S9.** Coverage-GC plots of contig properties for strain OF001 and A210. a, c) Coverage vs GC content, and b, d) coverage vs length. Both samples has a primary, high abundance cluster of contigs, with GC centered around 0.6 with arise form the primary culture populations. For strain OF001 a second contig cluster with GC centered around 0.35 at a much lower abundance was detected, which might represent a slight DNA contamination. **Table S1.** List of genes in strains OF001 and A210 with homology to putative Mn^2+^ oxidases from other MOB. **Table S2.** Functional domains and ontology classification of MO-mco and MO-hpox from MOB. **Table S3.** List of sequences of MO-mco and non- Mn^2+^ oxidases. **Table S4.** List of genes related to the metabolic potential of *Pseudomonas* sp. OF001 and *Rubrivivax* sp. A210. **Table S5.** List of cytochrome genes within *Pseudomonas* sp. OF001 and *Rubrivivax* sp. A210. **Table S6.** List of genes related to cell motility and biofilm formation within *Pseudomonas* sp. OF001 and *Rubrivivax* sp. A210. **Table S7.** List of genes related to degradation of organic compounds in *Pseudomonas* sp. OF001 and *Rubrivivax* sp. A210. **Table S8.** Genomic islands identified within *Pseudomonas* sp. OF001 and *Rubrivivax* sp. A210 genome sequences. **Table S9.** Characteristics of prophage regions identified in *Pseudomonas* sp. OF001 and *Rubrivivax* sp. A210 genome. **Table S10.** CRISPR-Cas systems detected within *Pseudomonas* sp. OF001 and *Rubrivivax* sp. A210 genome. **Table S11.** Characteristics of the *cas* genes detected within *Pseudomonas* sp. OF001 and *Rubrivivax* sp. A210 genome. **Table S12.** Comparison of repeats in the CRISPR of confidence level 4 found in strain OF001 to CRISPRCasdb. **Table S13.** Comparison of spacers in the CRISPR of confidence level 4 found in strain OF001 to CRISPRCasdb. **Table S14.** Comparison of repeats in the CRISPR of confidence level 4 found in strain A210 to CRISPRCasdb. **Table S15.** Comparison of spacers in the CRISPR of confidence level 4 found in strain A210 to CRISPRCasdb.

## Data Availability

The data for this study have been deposited in the European Nucleotide Archive (ENA) at EMBL-EBI under project number PRJEB40009 with accession numbers GCA_904426495 and GCA_904426505 for strain OF001 and A210, respectively.
