# Supplementary material for: Genome analysis of Pseudomonas sp. OF001 and Rubrivivax sp. A210 suggests multicopper oxidases catalyze manganese oxidation required for cylindrospermopsin transformation
Source: BMC Genomics. 2021 Jun 22;22:464. doi: 10.1186/s12864-021-07766-0 (PMC8218464; doi:10.1186/s12864-021-07766-0)
Supplement: Supplementary file 1 — Additional file 1: Fig. S1. Phylogenetic tree based on 16S rDNA sequences and whole genome sequences including strain OF001 sequence. Tree inferred with FastME 2.1.6.1 [129] from GBDP distances calculated from a) 16S rDNA gene sequences and b) genome sequences. The branch lengths are scaled in terms of GBDP distance formula d5. The numbers above branches are GBDP pseudo-bootstrap support values > 60% from 100 replications, with an average branch support of a) 68.8% and b) 92.5%. Tree was rooted at the midpoint [163]. Bold text represent the sequences generated in the present work. Scale bar represent sequence divergence. Fig. S2. Phylogenetic tree based on 16S rDNA sequences and whole genome sequences including strain A210 sequence. Tree inferred with FastME 2.1.6.1 [129] from GBDP distances calculated from a) 16S rDNA gene sequences and b) genome sequences. The branch lengths are scaled in terms of GBDP distance formula d5. The numbers above branches are GBDP pseudo-bootstrap support values > 60% from 100 replications, with an average branch support of a) 76.8% and b) 83.4%. Tree was rooted at the midpoint [163]. Bold text represent the sequences generated in the present work. Scale bar represent sequence divergence. Fig. S3. Pan- and core genome overview. Venn diagram shows the number of shared and specific Microscope gene families (MICFAM) a) among Pseudomonas sp. OF001 and the members of the Pseudomonas_K group, and b) among Rubrivivax sp. A210 and the members of the Rubrivivax genus. MICFAM grouping was based on 50% amino acid identity cut-off and at least 80% amino-acid alignment coverage. Fig. S4. Pan- and core- genome sizes estimated evolution. a, c) Number of MICFAM families in the pan-genome size by the number of genomes, and b, d) number of MICFAM families in the core-genome by the number of genomes. a, b) Including Pseudomonas sp. OF001, and c, d) including Rubrivivax sp. A210. Fig. S5. Maximum Likelihood phylogenetic tree based on multicopper oxidases s [file 12864_2021_7766_MOESM1_ESM.zip › Table_S14.docx]

Table S14. Comparison of repeats in the CRISPR of confidence level 4 found in strain A210 to CRISPRCasdb.

| Repeat | Matched nucleotides | BLAST score bits | BLAST e-value | Organism | Accesion number | |
| --- | --- | --- | --- | --- | --- | --- |
| ACTTCAACGATGCCGCGCCCAAAGGGTGCGGTGAG | n.m. |  |  |  |  |  |
| GCTTCAACGATGCCGCGCCCCAAGGGCGCGGGGAA | n.m. |  |  |  |  |  |
| GCTTCAACGATGCCGCACCCCATGGGCGCGGTGAG | 19/36 | 32.2 | 0.002 | *Verrucomicrobium spinosum* | ABIZ01000001.1_7 |  |
| ACTTCAACGATGCCGCGCTTCAAAGGCGCGGTGAG | n.m. |  |  |  |  |  |
| ACTTCAACGATGCCGCGTCCCAGGGGCGCGGTGAG | n.m. |  |  |  |  |  |
| GCTTCAACGATGCCACGCCGCATGGGCGTGGTGAG | 20/36 | 34.2 | 0.0005 | *Opitutaceae* bacterium TAV5 | CP007053.1_2 |  |
| GCTTCAACGATGCCGCGCCACAAAGGCGCGGTGAG | n.m. |  |  |  |  |  |
| GCTTCAATGATGCCGCGCCTCAAAAGCGCAGTGAG | n.m. |  |  |  |  |  |
| GCTTCAACGATGCCGCGCCACAAAGGCACGGTGAG | n.m. |  |  |  |  |  |
| GCTTCAACGATGCCGCGCCTCAAAGGCACGGTGAG | n.m. |  |  |  |  |  |

n.m.: no matches
